# Army Veterinary Service1Read at the Thirty-sixth Annual Meeting of the American Veterinary Medical Association, New York, September, 1899.

**Published:** 1899-09

**Authors:** Rush S. S. Huidekoper

**Affiliations:** (Univ. of Penna.), Veterinarian, Republic of France; Late Lieut.-Col. and Chief Surgeon, First Army Corps


### THE JOURNAL OF COMPARATIVE MEDICINE AND VETERINARY ARCHIVES.

VOL. XX. *SEPTEMBER, 1899.* No. 9.

### ARMY VETERINARY SERVICE.[1]

BY RUSH S. HUIDEKOPER, M.D. (UNIV. OF PENNA.),
VETERINARIAN, REPUBLIC OF FRANCE; LATE LIEUT.-COL. AND CHIEF SURGEON, FIRST ARMY CORPS.

MR. PRESIDENT AND GENTLEMEN: While in Europe, in 1881–1883, studying the organization of the veterinary schools of the various countries, I became interested in the veterinary service of their armies, both from the direct connection of the veterinary schools of Continental countries with their war departments and from my interest in military affairs as a medical officer of the National Guard of Pennsylvania. I then realized the importance of the subject, and in establishing the Veterinary Department of the University of Pennsylvania I projected in the original plans of that school and put into execution courses of instruction which had not existed in America, but which had a direct bearing on the education of veterinarians for army and other governmental service. The forges for practical farriery were put in operation, the course of meat-inspection was commenced, and the plans of the institution included large cattle-stables and a school of equitation. From that time for several years, as a member of this Association, I was a member of the committees attempting to obtain legislation for the recognition of veterinarians in the United States Army. During that time I had the honor and pleasure of gaining the personal friendship of General Philip Sheridan. That great cavalry officer, who had become Lieutenant-General of the army, took an immediate interest in the subject, and the year preceding his death he had a scheme of organization prepared by Colonel Sandford C. Kellogg (an aid on his staff) and myself, which he corrected in form to be presented to the next Congress. General Sheridan's death caused a serious delay to the foundation of a proper veter-

[1] Read at the Thirty-sixth Annual Meeting of the American Veterinary Medical Association, New York, September, 1899.

inary service in our army, for his interest and personal influence would undoubtedly have led to the establishment of such a service at that time. The following two winters I was still on the committee of this Association, and spent a great deal of time in Washington. General Schofield approved the organization of a much smaller veterinary corps than General Sheridan had provided for, and its chance of success was very encouraging until an opposition sprang up, engendered by the veterinary surgeons employed by civil contract in the cavalry regiments, who violently fought every measure which would require an examination of competency from them. Among these at that time were men who could scarcely read and write.

For the following few years our Association kept steadily at work, with little result until the last session of Congress. During this time circumstances prevented me from personally following the details of the work done until last winter, when I was located in Washington and was glad to aid Dr. D. E. Salmon and the committee in its work. The result of last winter's work has been given in the veterinary journals, and is presented at this meeting in the President's address and in the report of the Committee on Army Legislation. It may be summarized as follows :

The Hull Bill (H. R. 11022) in the House of the recent Congress provided "two veterinarians" for each regiment of cavalry, as follows :

The veterinarians provided for in this section shall have the rank, pay, and allowances of a second lieutenant of cavalry : *Provided*, That the veterinarians provided for above shall stand a competitive examination to be held under rules and regulations to be prescribed by the Surgeon-General of the United States : *And provided further*, That the veterinary surgeons now in service with cavalry regiments may be appointed as veterinarians without reference to age : *And provided further*, That in computing length of service for retirement such veterinarians shall be credited with the time they have already served as veterinary surgeons.

The Senate sub-committee replaced this section, which was accepted by the Republican half of the Senate committee of military affairs, with the following :

Of the veterinarians provided for in this Act one shall have the pay and allowances of a second lieutenant of cavalry, and one with the pay of seventy-five dollars per month and the allowances of a sergeant-major : *Provided*, That the veterinarian appointed to the first grade shall not be so appointed until he shall have passed an examination, to be prescribed by the Secretary of War, as to his mental, moral, and professional qualifications : *Provided further*, That the veterinarians now in the service who do not pass such competitive examination shall be eligible to the positions of the second class under such rules as are now prescribed by the regulations. The regimental sergeant-major and the regimental quartermaster-sergeant provided for in this section shall have the pay and allowance of an ordnance sergeant.

At this time, after consultation with Dr. D. E. Salmon, I placed the matter before the President, the Secretary of War, Major-General Miles, and the Quartermaster-General, and submitted to them a draft of an amendment for the organization of a veterinary corps, to replace the provisions of the Hull Bill or the Senate amendment, which only provided for the employment of veterinarians in the cavalry.

The President, without hesitation, said that he would approve of the organization of a veterinary corps for the army. The Secretary of War, Russell A. Alger, promptly indorsed in writing an amendment with these words: "*I approve of the organization of a veterinary contingent to the army.*"

General Miles expressed great interest and approval of such an organization, and said that he would approve any legislation on the subject which should be submitted to him.

Senator Kenney, of Delaware, kindly offered to prepare the amendment, which he presented and went actively to work for. This amendment read as follows:

Amendments intended to be proposed by Mr. Kenney to the bill (H. R. 11022) for the reorganization of the Army of the United States and for other purposes, viz.:

On page 1, in line 11, after the word "corps," insert the following, "a veterinary corps."

On page 2, in line 13, strike out the words "two veterinarians."

On page 3 strike out all after the word "privates," in line 7, down to and including the word "regulations," in line 19.

On page 7, at the end of section 7, add the following:

"SEC. —. That the veterinary corps shall consist of a chief veterinary officer, with the rank, pay, and allowances of a colonel, United States Army; 10 veterinarians, each with the rank, pay, and allowances of a first lieutenant of cavalry; and 20 veterinarians, each with the rank, pay, and allowances of a second lieutenant of cavalry.

"The original vacancies in the force created by this section may be filled by selections from veterinarians who have served in the armies of the United States in the war with Spain or from civil life: *Provided*, That no person shall be appointed a lieutenant in this corps until he shall have passed a satisfactory examination as to his physical, moral, and professional qualifications before a board to be appointed by the Secretary of War, of which board the chief veterinary officer shall be one, whose proceedings and finding shall first be approved by the Secretary of War.

"All rules and regulations governing the veterinary corps shall be made by the Secrctary of War, and the chief veterinary officer shall report directly to that officer.

This amendment, having the approval of the President and General Miles and bearing the written approval of the Secretary of War, was read in the Senate, and a vote was called for by Senator Chandler, of New Hampshire; but the chairman recognized Mr. Vest, of Missouri, who gained the floor, and in the radical upsetting of the Army Bill which took place in the next few hours until final passage it was not possible to call for a vote again.

After the adjournment of the last Congress I again brought the matter before the President, the Secretary of War, General Miles, Senator Hawley, the chairman of the military committee of the Senate; Mr. Hull, the chairman of the military committee of the House, and many others—army officers, Senators, and Congressmen—and I am satisfied, from the interest and promise of support which has been given to me, that legislation establishing a veterinary corps can be secured.

While the large majority of persons who have been consulted have expressed their appreciation of the need of a veterinary service, both from its economic stand-point and on account of the increased efficiency which it will produce in the horses of the cavalry, artillery, and transportation of the army, few have proved familiar at all with the details of such an organization or its relation to the other branches of the army. Last winter I applied to army veterinarians in England and Continental Europe for such information, and received many interesting replies from England, France, Germany, Austria, Italy, and Belgium. Unfortunately, only a portion arrived in time to be used in the last Congress. I submit for record with this paper a copy of the circular issued by the Committee on Army Legislation and a copy of the JOURNAL OF COMPARATIVE MEDICINE AND VETERINARY ARCHIVES of March, 1899, with two articles marked. In these are summaries of the veterinary force of most of the European armies.

One of the last communications which I recently received from Europe is a letter and printed matter from Colonel C. M. Dubois, Chief Veterinarian of the Army of Belgium. As the establishment of a veterinary corps as a separate department of the War Office in Belgium is the last of those in Europe, and has had and taken advantage of the lessons taught by the others, I have selected Colonel Dubois' communication to present to you to show the nature of service of the army veterinarians in European countries.

[*Translation.*]

BRUSSELS, June 5, 1899.

LIEUTENANT-COLONEL R. S. HUIDEKOPER,

*Sir and Honored Colleague:* I received your letter of February 17th last at a moment when I was absolutely overworked. I had numerous reports to make to the Minister of War, and I had to preside over the sessions of examination for the military veterinarians. I was obliged also, to answer completely your request, to make for you a résumé of our regulations, for the Minister of War, to whom I applied on the subject, informed me that he had no extra copy.

The veterinary service of the army forms, since 1892, a service absolutely independent; the chief of the service finds himself directly in relation with the Minister of War without any intermediary. Before 1892 the veterinary service made part of the *Service of Health of the Army* ("Service de Santé de l'Armée"), which comprised the medical men, the pharmacists, and the veterinarians. The service was directed by a medical man—the Inspector-General of the Service of Health of the Army—having the rank of Major-General. The Chief Veterinarian was attached to the general inspection of the Service of Health, all measures of our service being made on its propositions. It was only on the question of veterinary scientific problems that the Chief Veterinarian intervened personally. To-day all the matters which interest the veterinary service are directed by the Chief Veterinarian, and all propositions come from him.

Our service comprises at present the Chief of Service: Chief Veterinarian (Lieutenant-colonel); twelve Chiefs of Service: two Principal Veterinarians (Majors); ten Regimental Veterinarians First-class (Captains). Twenty-two adjuncts to Chiefs of Service: Five Regimental Veterinarians Second-class (Captains—"en Second); nine Veterinarians Second-class (Lieutenants); eight Veterinarians Third-class (Sub-lieutenants).

Additional pay is allowed to captains of twenty-five years' service and to lieutenants of twelve years' service. All must mount themselves, but receive indemnity for forage. The Chief of Service has indemnity for expenses of Bureau.

The veterinary *personnel* comprises also, since 1889, assistant veterinarians up to the number of thirteen; they are divided into first and second class; the first are assimilated to the grade of Adjutant (sous-officier), the second to Maréchal des Logis Chef. After six months' service as assistant second class they can be proposed for the first class. The object of the creation of the assistants was to augment the *personnel* without apparently increasing the part of the Budget of War applying to the veterinary service. I deplored this innovation, which diminishes our prestige and discourages young men who desire to enter the army. It appeared to me that it would have been preferable to keep the number organized before; it would have sufficed to have given all service—our organic effectiveness of horses in peace-footing being only about 7300; in war-footing our number is augmented by all the civil veterinarians of the militia, who have the title of Auxilliary Veterinarians. I believe that those who were partisans of the assistants found in it the advantage of having a large number of adjuncts in order to get rid of a part of their work, and eventually to replace themselves, so as to occupy themselves more easily with their private practice; at the same time, in augmenting the *personnel* it seemed proper to claim a larger number of superior officers. I am not of this opinion. I consider that the grade and rank ought to be in relation to the importance of the duty and not on account of the numbers.

The former organization comprehended for each of the eight regiments of cavalry (effectif, 615 troop horses) one veterinarian, Chief of Service, one of second class, and one of third class; and for each of the four regiments of field artillery (of which there are two of 450 horses and two of 600 horses) a Chief Veterinarian and a second-class veterinarian. There was, moreover, a veterinarian of the second class for the regiment of transportation and one for the detachment of Gendarmerie of Brussels.

In a project of reorganization proposed by the Ministry and submitted at present to the Chamber of Representatives and to the Senate there will be: One Chief Veterinarian (Lieutenant-Colonel), four Principal Veterinarians (Majors), ten Regimental Veterinarians (Captains), five Veterinarians First-class (Captains—"en Second"), eight Veterinarians Second-class (Lieutenants), seven Veterinarians Third-class (Sub-lieutenants), six Adjuncts (Sub-lieutenants in appointments), and four Assistants (non-commissioned officers).

I would have preferred to have seen the rank of Colonel given to the Chief of Service and the corresponding advance to the others; *for from the point of view of military service it is indispensable that the Chief of the Veterinary Service should have the grade of the chief of the regiments whom he is expected to advise.*

In order to be admitted to the army one must have a diploma of veterinarian granted by the Government Veterinary School—the only one in Belgium—and be twenty-six years of age.

Since October 1, 1892, the assistants, on their entrance into service, follow a course of equitation of ten months at the Military School of Equitation of Belgium, at Ypres, with the division of sub-officers; they are subjected to the same physical exercises. At the same time they are initiated in the Military Veterinary Service and attend the lessons on horseshoeing given to the division of students of farriery. After one year of service the assistant undergoes Examination A, which he must pass to be nominated Veterinarian, third class (officer). It comprises :

1. Examination and treatment of two diseases, external or internal; rendering a complete report and discussion for an hour on the work.

2. Practique of two surgical operations, after having exposed the anatomy of the region, the different operating methods, consecutive care, discussion of one hour.

3. Solution in writing of two questions, at least, on (*a*) hygiene; (*b*) regulations, accounts, remounts, condemnation, etc.; (*c*) appreciation of the quality of forage, butcher animals, diagnosis of the diseases which render meat improper for food; (*d*) bacterial and parasitic diseases.

4. Examinations (facultative at present): Microscopical demonstrations, normal and pathological histology.

5. Oral examination of half an hour on some part of farriery.

6. Examination of the military part, comprising topography, military art, fortification, rights of men, regulations concerning service in garrison, field service, etc.

7. Practical proof in equitation.

Examination B. is required to be nominated Chief of Service (Regimental Veterinarian). For this one must have at least two years' grade of Veterinarian, second class. This examination comprises :

1. Examination and treatment of four horses affected with external or internal diseases; a written report, followed by a discussion of one hour.

2. Practique of four surgical operations after having exposed the anatomy; the different methods, and discussion of one hour.

3. Lecture on hippology of one hour. This proof is required because the Chiefs of Service (regimental surgeons) are required to give lectures on hippology to the officers of the corps to which they are attached.

N. B.—In our examinations all the questions are drawn by lot. The lecture on hippology proving an obstacle which caused the failure of many candidates, the Veterinary Inspector decided, when I took direction of the course at the School of Equitation, in 1868, to give to me successively all the veterinarians, second class, in order to instruct them completely in farriery, and to let them give the courses in hippology to the non-commissioned officers.

During five years successively I had a new assistant each year at the School of Equitation, and after this preparation all succeeded in their examinations, although several of them had failed before.

. . . . . . . . . . . .

When I was appointed to the direction of the Veterinary Service, in 1895, I re-established the system (in disuse) which had been inaugurated by M. Marcoux in 1872, and obliged all the veterinaarians, second class, to take a course at the School of Equitation.

The first year they follow the course of equitation—grooming, stabling, feeding, harnessing, fencing, gymnastics, the instruction in regulations (army), accounts, etc. They attend the courses of hippology and farriery. The second year they give instruction in the same to the divisions of non-commissioned officers and men under the direction of the professor.

The course of farriery necessitates in effect a man having considerable experience in both theory and practical horseshoeing. In this way, studying practically equitation and grooming, they acquire a serious knowledge of the horse, and are better able to judge the qualities which they ought to exact in a horse for service when they become in the regiments the useful counsellors of their own chief and of the officers whom they serve, and with the latter it gives them more prestige than the scientific knowledge they may have, which is little appreciated by the laymen.

I succeeded in convincing the authorities to carry out these measures, which have now been in full force for two years and have given magnificent results. But it exacts from the veterinarian an amount of work which he would like to be free from, and I meet with considerable resistance. I will add that I have myself followed during the first year the course in grooming, stabling, feeding, etc., while I gave the lectures to the officers. In exacting the application of the regulations concerning the making of reports, clinical bulletins, etc., I have discontented the chiefs of service accustomed to "far-niente."

. . . Opposition, uniforms, etc. . . .

Our farriers (horseshoers), who were simply privates, have been promoted to non-commissioned officers.

. . . Sending of army veterinary regulations, copies of blanks, etc. . . .

Please accept, sir and honored colleague, the assurance of my best fraternal sentiments.

(Signed) C. M. DUBOIS,
Chief Veterinarian of the Army of Belgium.

Colonel Dubois sent me under separate cover a printed copy of the "Regulations of the Veterinary Service" and a copy of his own work on hippology: *A Study of the "Cheval de Guerre."*

This letter of Colonel Dubois is a concise résumé of the information I had already obtained from other countries. In France the veterinarian who enters the army on leaving one of the three Government schools enters the Cavalry School at Saumur and has a year's instruction in military education before being assigned to a regiment. In England and Italy it is the same; in Germany and Austria many of the veterinary students are soldiers before entering the schools, and have to serve a long probation as non-commissioned officers before their final examinations, when they become officers.

I will not repeat what has been gone over in previous addresses and circulars of your committee—of the necessity of a separate corps instead of the individual veterinarians as they are now attached to cavalry regiments. The necessity is obvious. The artillery and the transportation animals of the Quartermaster's and other departments must be provided for.

The cadets at West Point should have a thorough course of instruction in hippology, so that when they receive their commissions as officers and become responsible for the horses of a troop of cavalry or a battery of light artillery they should know something —as much as a layman can commence with—of the conformation of a horse, its physiology, stable hygiene, nutrient value of forage, and last, but not least, the care of the foot and shoeing.

The cavalry and artillery schools at Forts Riley and Leavenworth should have more advanced instruction for both officers and men, and at these posts there should be perfected schools of farriery, where every horseshoer in the army should receive in turn a minute, detailed course on the anatomy of the horse's foot, and a supervised term of practical application of the horseshoe. The cattle, bought for butchering for food for the army, should be inspected by army veterinarians who are known to be competent and responsible. The meat furnished to the army should be inspected by the same officers, who by education are the trained experts for such work. The forage fed to the army horse and mule should be inspected by the veterinarian, whose college education in botany and chemistry has fitted him for it.

Every board of officers appointed for the purchase of horses should have as one member an army veterinarian, an expert, trained in the details of faulty conformation and in the diagnosis of disease which will escape the observation of the cavalry or artillery officer, though the latter may, by long experience, be infinitely the superior and better expert in selecting the type of animal wanted for draught or saddle. While the professional veterinary work in any of these details can be performed by the employment of a civil veterinarian, as has been the custom in the Quartermaster's department and at many of the cavalry and artillery posts, this method is not satisfactory. It is more expensive, and the civil veterinarian is not responsible for the work beyond the moment of its performance. The employment and position of the veterinarian as provided for by the last Congress is little better. There has been gained the assurance of the employment of a better class of men by the requirement of the examination ; that is about all. The cavalry only is provided for, and the veterinarian is responsible only to the troop commander under whom he serves.

What is needed for an efficient service on the level with that of all other civilized countries is a corps, with the chief responsible directly to the head of the army, as are the Quartermaster-General, Surgeon-General, Chief Signal Officer, and others.

From the purchase of any horse or mule, cattle for the soldier's ration, or forage for the animals, a record should be forwarded through veterinary channels, so that, on the complaint of any troop or battery commander or commissary of subsistence through their channels, the chief veterinarian can locate the justice or error in the complaint. With a corps the chief will see that the veterinarians are equipped with proper supplies for performing their professional work ; he will be informed of the individual qualities of the personnel, and will be able to make the best selection of the individual veterinary officers for service, with troops in the field, at posts where instruction is required, and for details for boards of purchase and condemnation, and for scientific investigation of contagious diseases. The reports of the veterinarian on the condition of the animals for whose health he is responsible will coincide with the efficiency of the horses of a given command, or will furnish the information needed by the general commanding the army to explain inefficiency of any troop or battery.

Rank—the position of a commissioned officer—is a necessity to secure the services of a class of men who will be and will know themselves to be gentlemen, and who will have an esprit de corps which will compensate them like other army officers for the expenditure of intelligence and hard work far beyond that given in civil life for the money remuneration which an officer receives.

You, gentlemen, will have your share to do. When Congress grants us what we ask—a veterinary corps—the young men who pass the examinations and receive commissions will have no sinecure. The labors of the first veterinarians of such a service will be arduous and will require diplomacy and tact. A certain prejudice, justified in many ways, does exist in regard to veterinarians. You, to your scientific attainments, must add zeal, interest in your work, careful respect for your fellow-officers, and have your work at heart, and it will take but a few years' work to show the public that the veterinary corps will be the equal in value and esteem to any of the other departments of the army.

